# Hedgehog Signaling for Urogenital Organogenesis and Prostate Cancer: An Implication for the Epithelial–Mesenchyme Interaction (EMI)

**DOI:** 10.3390/ijms21010058

**Published:** 2019-12-20

**Authors:** Taiju Hyuga, Mellissa Alcantara, Daiki Kajioka, Ryuma Haraguchi, Kentaro Suzuki, Shinichi Miyagawa, Yoshiyuki Kojima, Yutaro Hayashi, Gen Yamada

**Affiliations:** 1 Department of Developmental Genetics, Institute of Advanced Medicine, Wakayama Medical University, Kimiidera 811-1, Wakayama 641-8509, Japan; hyuga520@wakayama-med.ac.jp (T.H.); mcalcant@wakayama-med.ac.jp (M.A.); 09p851kd@wakayama-med.ac.jp (D.K.); k-suzuki@wakayama-med.ac.jp (K.S.); 2 Department of Molecular Pathology, Ehime University Graduate School of Medicine, Shitsukawa, Toon City, Ehime 791-0295, Japan; ryumaha@m.ehime-u.ac.jp; 3 Department of Biological Science and Technology, Faculty of Industrial Science and Technology, Tokyo University of Science, Tokyo 125-8585, Japan; miyagawa@rs.tus.ac.jp; 4 Department of Urology, Fukushima Medical University School of Medicine, 1 Hikarigaoka, Fukushima 960-1295, Japan; ykojima@fmu.ac.jp; 5 Department of Pediatric Urology, Nagoya City University, Graduate School of Medical Sciences, 1 Kawasumi, Mizuho-cho, Mizuho-ku, Nagoya 467-8601, Japan; yutaro@med.nagoya-cu.ac.jp

**Keywords:** hedgehog, epithelial–mesenchymal interaction (EMI), prostate cancer, external genitalia, androgen, basement membrane, bone morphogenetic protein

## Abstract

Hedgehog (Hh) signaling is an essential growth factor signaling pathway especially in the regulation of epithelial–mesenchymal interactions (EMI) during the development of the urogenital organs such as the bladder and the external genitalia (EXG). The Hh ligands are often expressed in the epithelia, affecting the surrounding mesenchyme, and thus constituting a form of paracrine signaling. The development of the urogenital organ, therefore, provides an intriguing opportunity to study EMI and its relationship with other pathways, such as hormonal signaling. Cellular interactions of prostate cancer (PCa) with its neighboring tissue is also noteworthy. The local microenvironment, including the bone metastatic site, can release cellular signals which can affect the malignant tumors, and vice versa. Thus, it is necessary to compare possible similarities and divergences in Hh signaling functions and its interaction with other local growth factors, such as BMP (bone morphogenetic protein) between organogenesis and tumorigenesis. Additionally, this review will discuss two pertinent research aspects of Hh signaling: (1) the potential signaling crosstalk between Hh and androgen signaling; and (2) the effect of signaling between the epithelia and the mesenchyme on the status of the basement membrane with extracellular matrix structures located on the epithelial–mesenchymal interface.

## 1. The Basic Architecture of Hedgehog Signaling

Hedgehog (Hh) signaling is an effective and critical regulatory growth factor signal for organogenesis. There are three ligands found in mammals: Sonic hedgehog (Shh), Indian hedgehog (Ihh), and Desert hedgehog (Dhh). Among these three, Shh is the best studied Hh ligand. During development, the expression of Shh is found often in the epithelia [1,2,3]. In addition to its ligands, various genes involved in the Hh signaling pathway, such as its receptors and transcription factors, have been identified [4]. Ptch (Patched) is a transmembrane receptor that functions as the primary regulator of Hh signaling [5,6,7,8]. In the absence of a Hh ligand, Ptch localizes to the base of the primary cilium, the center of Hh signaling in mammals, and prevents the movement of Smo (Smoothened), another membrane signaling component, into the primary cilium. The accumulation of Smo within the primary cilium results in its activation and the processing of downstream mediators. Thus, in the absence of a Hh ligand, Ptch blocks the Hh signaling pathway activation by inhibiting Smo. Therefore, it is the critical step to initiate the signaling cascade.

The downstream effects of the Hh signaling pathway are mediated by the Gli family of transcription factors [9,10,11,12]. This family consists of three major regulators: Gli1, Gli2, and Gli3. Gli1 is the transcriptional activator (Gli-A), while Gli2 and Gli3 can be processed into both activator and repressor forms (Gli-R). The accumulation of activated Smo suppresses the generation of Gli-R forms and allows Gli-A proteins to translocate into the nucleus and bind to their target genes. Thus, the canonical Hh signaling pathway is noted with Hh ligand binding to the membrane receptor, which activates Smo regulating the Gli transcription factors.

The Hh ligands are secreted molecules, often from the epithelia. These signals can be received by the neighboring mesenchymal layer, and the presence of both negative and positive regulators of the signaling pathway has been reported in these signal-receiving cells. This establishes a signaling crosstalk between the epithelia and mesenchyme, known as epithelial–mesenchymal interactions (EMI), which drives cell proliferation, differentiation, migration, and other developmental processes. The role of Hh signaling in EMI in both development and cancer will be discussed below.

## 2. Hedgehog Signaling and EMI in Urogenital Organogenesis

Loss of Hh signaling during mammalian development results in several defects such as polydactyly, cyclopia, and limb malformation, indicating the essential functions of Hh signaling pathway in organogenesis. In several cases, other growth factor signals are also involved in Hh signaling regulation during development. These factors are released from either the epithelia or the mesenchyme, depending on the developmental context. Urogenital organ development is a dynamic process, involving several signaling pathways which changes depending on tissue contexts. The urogenital system includes the urinary tract: composed of the kidney, the ureter, the bladder, and the urethra; and the reproductive tract: composed of the testes, the accessory glands, and the external genitalia (EXG). Recently, an increasing number of regulatory genes for EXG development has been reported [13,14,15,16,17]. Thus, possible insights gleaned from comparisons between urogenital organogenesis with other medical topics, such as prostate cancer, is valuable. This review offers such perspectives based on the recent findings for these topics.

During urogenital tissue formation, especially in the EXG, Hh signaling and its role during EMI is essential. One of its ligands, Shh, is expressed in the endodermal epithelia of the embryonic urethra [18,19]. The Shh released from the epithelia can regulate the differentiation of the bilateral mesenchyme, the tissue located immediately adjacent to the urethra [15,20,21]. Such regulation of the mesenchyme by epithelial signals is a key event for EXG organogenesis, as loss of this signal leads to the agenesis of the EXG anlage, the genital tubercle (GT) [19]. The interactions between this epithelial signal and the mesenchyme has been investigated in the development of several embryonic regions and stages – from the cloaca in the early stages of development (~E10.5) and later (from ~E13.5) in the bladder, the urethra, and the EXG [22]. The role of Hh signaling in EMI in late-stage, androgen-dependent formation of the EXG, including the formation of the male-type urethra, will be discussed below.

Several works report on the role of Shh signaling and EMI during urogenital tract development. In these organs, the Hh ligands are typically expressed in the epithelia; and the expressed growth factor ligands emanate signals towards the mesenchyme, which, in turn, relays signals to the epithelia. This can induce cellular proliferation or differentiation and, in some cases, may also result in the expression of other growth factor signaling genes. Such signaling between the two tissue layers constitutes an essential crosstalk during organogenesis. The growth factor ligands that have been reported to be responsive to Hh include BMP (bone morphogenetic protein), fibroblast growth factor, and Wnt signaling [18,23,24,25,26]. To fully understand the essential functions of Hh signaling, investigation of interacting signaling pathways is also necessary. Among them, some BMP ligands are expressed in the developing mesenchyme of the urogenital tract [26,27,28]. Several signaling studies have reported on the vital role of BMP signaling in mesenchymal differentiation during ureter and bladder smooth muscle development [27,29,30]. In the ureter, this BMP-expressing mesenchyme will form ordered layers with different type of cells, including the lamina propria and the surrounding smooth muscle cells [31]. One of the effective strategies to study the role of BMP signaling is to analyze the corresponding mutant mouse models for such signaling genes. Introduction of loss of function mutations of the Bmp signal-component genes in both ureteric and bladder mesenchyme led to impaired development such as hypoplasia of the smooth muscle [31,32]. Thus, proper BMP signaling has been shown to be essential in urogenital tract development.

Because Shh is expressed in the developing cloacal and ureteric epithelia, the interaction between epithelia-derived Hh signal and corresponding BMP signaling from the nearby mesenchyme was investigated [22]. To analyze this interaction, studies were performed on Gli1-expressing mesenchyme in response to Hh ligand simulation. *CreER*^*T*2^ is a tamoxifen inducible Cre recombinase gene, and it is knocked into the Gli1 gene locus by homologous recombination. Thus, taking advantage of the inducible nature of Gli1 gene expression, gene modulation in the target mesenchyme cells was performed using a Gli1-*CreER*^*T*2^ modifier mouse strain [22]. Following activation of Hh signaling, BMP signaling increased in the bladder, suggesting that BMP signaling is a downstream event to Hh [27,33]. BmprIa is the major Bmp type I receptor. It is expressed on the cell surface and interacts with the type Ⅱ receptor. Gli1-mediated gene knock-out of BmprIa led to hypoplastic ureteral smooth muscle formation [27]. As a result, the mutant mouse develops hydronephrosis, a severe urogenital phenotype [27]. Thus, signaling relays from Hh, particularly Shh, toward BMP signaling has been suggested. However, contradictory reports have also been published about such interactions. Addition of Shh to cultured neonatal prostate glands increased Bmp4 expression in the adjacent mesenchyme; however, addition of Noggin, the Bmp antagonist, did not override the Shh-induced growth inhibition [34]. This suggests that the increase of Bmp4 does not mediate the effect of Shh signaling. Further investigations of the downstream cellular functions using conditional mutant mice and other in vivo systems are necessary. The urogenital developmental process, therefore, offers an intriguing research opportunity for understanding EMI between Hh and its interacting signal cascades (Table 1).

## 3. Hedgehog Signaling and EMI in Prostate Cancer Tumorigenesis

Hh signaling has also been often implicated in tumorigenesis. Over the recent years, prostate cancer (PCa) has been recognized as one of the life-threatening cancers in men. PCa arises in the prostatic epithelia, and it has been reported to interact with the nearby mesenchyme [35,36,41]. In the adult tissue, the expression of the Hh signaling gene is not prominently detected, but it has been found to be present in the regenerating prostatic epithelium [58]. Considering this, the involvement of Shh in PCa tumorigenesis has been suggested. Shh expression has been frequently correlated with a higher Gleason score, the indicator of malignant type PCa [59,60,61]. In fact, Shh overexpression induced the growth of the tumor, the LNCaP cells, in a mouse xenograft model [35]. Furthermore, Shh has been reported to be highly correlated with the recurrence of the prostate specific antigen (PSA), a protein that is highly expressed in PCa [61]. The expression of Hh signaling components, such as Gli, is also correlated with a higher Gleason score [59,60,61]. During prostate development, Gli signaling is involved in the differentiation of prostate progenitor cells [62], and Gli mediates the oncogenic transformation of prostate basal cells [63]. Furthermore, Gli1 confers basal-like characteristics onto LNCaP cells, leading to the acquisition of PCa hormone independence [64]. Lastly, it has been reported that Hh signaling through Gli can support androgen signaling in both androgen-deprived and androgen-independent PCa [39,65]. Thus, experimental manipulation of Hh signaling leads to the oncogenic transformation of PCa.

Several studies have been published on the autocrine and paracrine modes of Hh signaling. As Hh signaling proteins can either be expressed solely in the epithelia, or in both epithelia and mesenchyme, there is still no strong consensus on the primary type of Hh signaling in PCa [61,66]. There is a growing concern on the possibility of EMI in PCa progression, as it will provide new information for the development of its treatments [66]. It is possible that Shh released from the prostatic epithelia can affect the nearby mesenchyme and modulate proliferation and differentiation [35]. This interaction may eventually promote PCa tumorigenesis. In addition to paracrine signaling, increasing attention has recently been given to autocrine Hh signaling in PCa [38,66,67]. It has been suggested that some PCa cells change from paracrine type of Hh to autocrine Hh, therefore giving them the ability to produce and respond to the ligand.

Comparing paracrine Hh signaling in organogenesis and tumorigenesis may provide new insights for future research directions [68,69]. BMP has also been investigated as a candidate responsive signaling pathway in PCa. PCa cells are highly invasive towards bone tissue, a phenomenon referred to as bone metastasis [70]. This occurs most prominently in the spine, particularly the lumbar vertebrae [71,72]. The BMP protein was originally identified from bone extracts, suggesting it as a candidate growth factor signal in the local environment. In such PCa metastatic sites, bone-derived signals such as BMP may play a role in modulating PCa malignancy. To investigate bone metastasis, bone-derived cell lines, such as osteoblast cells, were co-incubated with PCa cell lines, such as LNCaP, in experimental systems. Such in vitro systems enabled researchers to investigate the effects of soluble factors for cellular interactions, mimicking the metastatic site of PCa in bones [37]. However, aside from osteoblasts, there are also osteoclasts, fibroblasts, and immune cells present at the metastatic bone sites in vivo, reflecting the complex conditions of the invasive nature of PCa. This merits the use of other experimental assays for investigating the mechanism of bone metastases in PCa. This has led to the development of xenograft models. In this system, the xenograft, which contains a set of cancer cells, can be grafted into a host mouse and analyzed in vivo, simulating the cancer microenvironment. The expression or inhibitory function of the BMP antagonist, Noggin, has been implicated in such xenograft model experiments [41]. In this study, the authors overexpressed Shh in an LNCaP xenograft and found increased BMP7 expression in the neighboring environment, even in the presence of Noggin [41]. The role of Noggin in PCa has yet to be elucidated.

In addition to the above investigations, application of recent analytical techniques may be required. Many PCa tumors contain various types of cancer cells. The mesenchymal cells adjacent to the PCa cells are often termed as cancer associated fibroblasts (CAF). Interactions between PCa and CAFs has been noted to be essential for PCa pathogenesis [36]. This interaction can be regarded as a form of EMI, with the PCa taking the role of the epithelium. It is also noteworthy to mention the heterogeneity of CAFs in PCa [73]. Furthermore, some types of PCa cells are also known for circulating tumor cells [74]. Hence, single-cell level analyses could be a suitable approach to analyze the interaction between PCa and CAF.

In addition to such experimental models, information on the dysregulation of BMP signaling in some metastatic sites is available. Changes in the expression levels of several BMP ligands and BMP receptors have been implicated in bone metastasis [44]. BMPRs are typically expressed in the epithelia in normal tissues, but it has been reported that BMPRIA, BMPRIB, and BMPRⅡ are all down-regulated with increasing prostate malignancy [46]. A separate group also reported that there is a lower expression level of BMPRIB after androgen withdrawal in both cancer-bearing prostate glands and PCa cell lines [45]. As for the mode of release of BMPs in the tumor environment, some BMP ligands have been reported to be released from PCa. In bone metastasis experimental systems, bone-derived BMP7 and PCa-derived BMP4 functions have been reported [43,44,75,76]. Furthermore, loss of BMP2 tends to be associated with an increase in Gleason score [42].

Locally released BMPs and its relationship with Hh signals can also be noted in other reproductive organ tumors, such as ovarian cancer. BMP signals have been reported to function in cell differentiation in the ovary, particularly in the maturation of ovarian follicles [77] and has been reported to play a role in ovarian cancer pathogenesis [78,79]. Similarly, Hh has also been implicated in ovarian cancer progression [80]. All three type of Hh ligands—Dhh, Shh, Ihh—are expressed in ovarian cancers. Dhh expression, in particular, has been suggested to be correlated with its poor prognosis [81]. Interestingly, the presence of ovarian cancer-derived Hh signals and BMP4 from the surrounding mesenchyme has been reported [82]. Furthermore, they report that both Shh and Ihh form a signaling loop with BMP4, leading to chemotherapy resistance [82]. Thus, the importance of Shh has been reported in this system.

**Figure 1:**
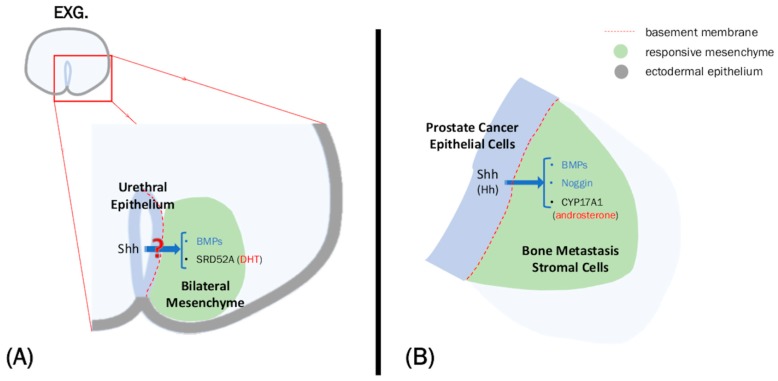
Overview of the role of hedgehog (Hh) in external genitalia (EXG) organogenesis and prostate cancer (PCa). (**A**) Sonic hedgehog (Shh) is expressed in the urethral epithelium and possibly regulates genes expressed in the mesenchyme. The result of this interaction may contribute to the masculinization of the EXG. (**B**) Similarly, Hedgehog ligands, particularly Shh, is expressed in the PCa epithelium and can promote PCa progression by regulating the expression of genes, such as bone morphogenetic protein (BMP), in the stroma. Shh can also promote the backdoor androgen pathway through CYP17A1 up-regulation.

Overall, various Hh ligands and its signals have been detected in the epithelia in both urogenital organ development and reproductive organ cancers, such as PCa and ovarian cancer. BMP is often expressed in the mesenchyme of these systems, and signals between these two tissue layers are important for both development and pathogenesis. This putative Hh-Bmp interaction and its role in cancer can be an interesting research topic, based on the transduction by the epithelial Hh signal interpreted at the level of the adjacent mesenchyme during urogenital organ development (Table 1, Figure 1).

What kind of consequences are expected as a result of such signaling crosstalk? The promotion and inhibition of PCa cell proliferation has been investigated in the field of cancer biology, but whether such environment-derived BMP render positive or negative effects for PCa cell proliferation requires further investigation. Recent studies indicate that the addition of recombinant BMP protein inhibits PCa growth in vitro [83]. Additionally, the effect of this crosstalk in cellular growth has also been analyzed. In general, both positive and negative regulation of proliferation are incorporated during developmental programs [24].

## 4. Modulation of Hedgehog Signaling by Androgen, the Male Hormone

Recently, progress on the mechanism of sexual differentiation has been described [84], and sexual differentiation with hormonal regulation is given much attention. The hormonal system has been classically described as a remotely acting biological system, as the signal emanates from the central nervous system and affects the target organs through the bloodstream. Testosterone, a gonadal hormone, is produced in the embryonic testes under the control of the central nervous system and regulates the masculinization of the reproductive tract, including the EXG anlage (GT) and other organs [85,86]. The definition of hormonal effects has been recently modified and extended to include their local action and production. These local actions have been suggested to participate in EMI [87], and recent evidence also suggests local modulation of hormone production. Hence, the possible significance of the interaction between local hormone signaling and growth factors is a growing concern. In this section, the relationship of the male hormonal system with the local growth factor, Hh signal, is discussed in the context of urogenital organ development and PCa.

In mice, EXG development occurs independently of androgen during the early stages. In the male, this is subsequently followed by an androgen-dependent masculinization process [15,88,89]. Robust mouse embryonic GT outgrowth is promoted by testicular androgens, starting from mid-gestation (~E14.0–15.0) until after birth [90]. The development of the male GT continues with the formation of a tubular urethra, a well-developed prepuce, and the condensation of a bilateral prospective corporal body. The male urethra is incorporated into the glans. This entire process is mediated by androgen, and these effects are generally described as consequences of “positive” androgen actions. Proper development of the male urethra enables efficient ejaculation during copulation and physiological reproductive male functions. In contrast to male development, the female GT does not form a tubular urethra. Thus, urethral formation is a useful landmark for investigating the mechanisms of masculinization and androgen-dependent signaling cascades.

Androgens, such as testosterone and 5α-dihydrotestosterone (DHT), are produced locally in both embryonic and adult tissues. Both androgens possess essential roles for GT organogenesis. In fact, androgen administration is used during treatment of several conditions in human patients. As administration of androgen can promote penis elongation, it is used as a treatment for micropenis [91]. Similarly, urologists administer androgen to boost the outgrowth of a patient’s penis before operating on conditions such as hypospadias [92,93]. A recent study has suggested that DHT can negatively regulate cell proliferation in the ventral side of the GT during urethral formation [20]. The enzyme which converts testosterone into DHT, type II 5α-reductase, is expressed in the bilateral mesenchyme prior to male-type urethral formation. This enzyme is encoded by SRD5A2 [20]. Direct measurement analysis using mass spectrometry revealed the differential distribution of DHT. Data showed that there is a higher production of DHT in the ventral side of male GT [94], which is the same region of the bilateral mesenchyme showing reduced rate of cellular proliferation. Thus, higher levels of locally converted DHT can reduce cell proliferation, an event necessary for male-type urethral formation. Although DHT-target regulators for organogenesis have yet to be identified, MafB has been reported to be a DHT-responsive gene for urethral formation [95,96]. Therefore, it is possible that the regulated production of DHT leads to reduced cell proliferation in the bilateral mesenchyme under the control of transcription factor MafB, which regulates male-type urethral formation.

The regulation of cell proliferation in the different stages of PC by androgens has been reported. In contrast to the developmental process, PCa progression is initially dependent on androgens but may switch into androgen-independent during the advanced stages. It has long been established that the proliferation and tumor growth of PCa is driven by androgen [97,98]. LNCaP cells proliferated in response to the concentration of DHT in vitro [99]. Furthermore, after injecting LNCaP cells in nude mice, the frequency of tumor development was significantly higher in the male versus female mice [99], indicating that the presence of androgens is necessary for tumor growth. Several other studies also report on the regulation of cell-cycle genes by androgen in other PCa models [100,101] and in normal conditions [102]. In both cases, androgen increased the expression of cyclins and their kinases while decreasing the expression of its inhibitors. This is further evidence of the proliferative effect of the androgens towards PCa. Similar to the developmental context, however, there are reports of inhibitory activities on PCa cell proliferation by androgen. In human castration-resistant prostate cancer (CRPC), addition of DHT did not usually exhibit a positive effect on cellular proliferation but instead inhibited it [103].

Due to the dependence of tumor growth on androgens, castration and administration of androgen signaling inhibitors are frequent treatments for PCa [104]. Although most cases respond positively to androgen deprivation, some cases proceed to a more lethal form of PCa. This stage, known as CRPC, is often associated with bone metastasis and has been reported to progress despite approximately castration levels of androgens. The formation of CRPC is reported to occur either through the androgen receptor (AR) or independently of AR. The AR is a nuclear receptor, which, upon binding its ligand, mediates downstream signaling. AR is reported to be present in CRPC tumors. In fact, it has been reported that AR is often overexpressed or mutated in CRPC [105,106]. AR levels has been reported to be higher in CRPC xenografts compared to primary tumors or benign prostatic hyperplasia [106]. This increase in AR has been suggested to increase sensitivity of the PCa to low levels of androgens, thus allowing the cancer to progress further.

Changes in steroid levels due to local steroidogenesis has been reported in CRPC using mass spectrometry [107], and this has been suggested as another mechanism by which CRPC progresses in a low-androgen environment. In the backdoor pathway, DHT is derived from androstenedione, an intermediate in the classical androgen biosynthesis pathway [49]. Gene expression analysis has confirmed the presence these enzymes and intermediates in PCa sites [49], and addition of androstenedione to androgen-independent PCa lines significantly enhanced tumor growth [49]. Also, the conversion of androgens via the backdoor pathway has been reported to induce cell proliferation [108]. Furthermore, androgen deprivation has been reported to promote DHT production via the backdoor pathway in PCa [109]. The backdoor pathway was first reported in the development of the prostate and testes in marsupials [110,111]. Since then, it has also been reported in mice [112,113] and humans [50,113,114]. It has recently been suggested that backdoor androgens play a role during masculinization. During human reproductive development, it has been reported that DHT levels in the fetal circulation and fetal testes are low. Instead, androsterone, an intermediate in the backdoor androgen synthesis pathway, is the major androgen within the circulation. It is, however, not expressed in the fetal testes but was instead detected in non-gonadal tissues such as the placenta. Furthermore, androsterone and testosterone are the only androgens that is higher in the male than in the female fetal circulation [50]. Taken together, this suggests that masculinization is dependent not only on the fetal testes, but also on other tissues capable of locally producing androgens. As DHT is low in the fetal testes, it is also suggested that androsterone may be converted into DHT within the GT [50]. Further studies related to the production of DHT and the GT are necessary.

Of note, it has also been suggested that paracrine type Shh signaling is involved in the local steroidogenesis in PCa [47]. Although it is generally believed that local steroidogenesis occurs within the tumor, there have been several reports stating that the stroma is also capable of steroidogenesis [47,48,115]. Steroidogenesis related with Hh signal in the mesenchyme of bones is also discussed in such works. This may be recognized as a form of EMI between Hh signaling and androgens in the cancer environment (Table 1, Figure 1). Whether this paracrine interaction between growth factor signals, such as Shh, and hormonal signals occurs during organogenesis is still unclear. Modes of paracrine action may differ from stage to stage in organogenesis, as well as in PCa status. Hence, the role of Hh and androgen signaling in development and cancer may be discussed not only in the context of EMI but also in the chronological context.

Androgen signaling is mediated by the nuclear androgen receptor AR. Gli1, the major Hh downstream transcriptional regulator, may interact with AR in some types of PCa cell lines [39]. BMP signal is transduced in the responding cell by pSmad signaling. There are some studies indicating the presence of pSmad–AR interactions in the responding cell nucleus [116]. Several other cofactors have been reported to interact with AR and pSmad, respectively [116]. The involvement of male hormones and its potential crosstalk with Hh and BMP signals in PCa progression and organogenesis requires further analysis.

## 5. The Status of the Basement Membrane (BM) As a New Aspect of EMI Regulation

Previously, the description of EMI has been limited to the cellular layers, but recently, the participation of the basement membrane (BM) in EMI has been suggested [56]. When the BM was first described, its primary function was stated as a structural protein that acts as a barrier or as an adhesion site. Since then, it has been revealed that the BM possesses a wide range of functions that is critical in development and cancer [54,117,118]. For example, one of the main BM proteins, laminin, can regulate various biological processes by directly activating signaling pathways through its interactions with integrin receptors [119,120,121]. More recently, the status of the BM, including BM protein deposition and the BM integrity, has been described as an emerging regulator of signaling related with the function of EMI.

The proper development of the BM is essential during organogenesis: its formation and subsequent removal is an essential step in the development of several organs. This removal is particularly essential in cases wherein there is tissue fusion in the midline region, such as in the formation of the male-type urethra and the palate. Morphological and loss of function studies on palatal fusion revealed the necessity of regulating the status of the BM during this process [122]. It has been suggested that soluble growth factor signals, such as TGF-β, may participate in regulating this process [123]. Moreover, the failure of the midline to fuse properly has been suggested to result in palatal cleft phenotypes. Hence, the attention to defects in the regulation of the BM components, which results in developmental abnormalities, has been increasing.

Shh is one of the important soluble factors involved in the maintenance of the BM during organogenesis. The BM is composed of several extracellular matrix proteins, including laminin and collagen. During urogenital organ development, loss of the α5 chain of laminin has been reported to result in the formation of an abnormal urethra [55]. As mentioned above, growth factor signals, including Hh, are essential in the regulation of urethral formation during early mouse development (E11.5–E14) [18,19,22]. Shh is prominently expressed in the embryonic urethral epithelium in the mouse, and functional disruption of Shh during early development (~E10.5) leads to GT agenesis [19]. Subsequent conditional gene knock-out studies for Shh in later stages (E11–13.5) revealed that Shh is essential in urethral formation, as such knock-out models showed a hypospadias-like phenotype [40]. Detailed gene expression analysis revealed that absence of Shh expression during later stages of urethral development (E15.5–16.5) might be associated with the reduced level of laminin in the BM [Alcantara, unpublished]. In fact, Shh has been shown to be necessary for laminin deposition and BM formation in the myotome [124]. Thus, the loss of laminin may indicate that Shh signaling is required for the maintenance of BM structure in the prospective fusion site in the murine embryonic urethra. The effect of Shh on laminin regulation, however, during male-type urethral fusion should be analyzed further.

How the BM participates in EMI is a growing research field. As the disappearance of the epithelia is associated with the breakdown of the BM, it is likely that the changes in the BM are mediated, at least in part, by the epithelia. As mentioned, epithelial Shh can induce the deposition of laminin in the BM. Loss of Hh signaling results in defective myotome in both mice and zebrafish [57,124]. However, the possible role of the mesenchyme should not be discounted. In the zebrafish myotome, epithelial Shh-induced laminin deposition inhibited BMP signaling in the mesoderm leading to myotomal cell fate specification [57]. Thus, Hh from the epithelia may participate in paracrine signaling via the BM for the above organogenesis. In contrast, laminin can induce Shh in the hair follicle epithelia by triggering Noggin expression in the mesenchyme [56]. This evidence shows that the BM should be taken into consideration during EMI studies.

Another emerging possibility for the role of BM integrity in EMI is its potential to regulate protein diffusion between the epithelia and the mesenchyme. A study performed on MCF10A acini showed that the status of the BM is positively correlated with molecular permeability [125]. As the breast acini matures, the BM develops and thickens with it. Therefore, they compared the rate of permeation by dextran in low-matured and semi-matured acini. They revealed that in low-matured acini, the dextran penetrated the thin BM efficiently. On the other hand, semi-matured acini with thicker BM could significantly slow down permeation of the dextran [125]. Hence, it is possible that the changes in the BM integrity is responsible for allowing the diffusion of proteins, consequently influencing organogenesis. A similar case might occur in the urethra, where there is loss of BM integrity specifically at the fusion site of the embryonic urethral epithelia [Alcantara, unpublished]. Further investigation is necessary to establish such a role of BM in development.

Cell migration is a key event during metastasis, and it is important to consider the role of the BM during this event. The proper regulation and dysregulation of the BM can be related with PCa progression, as there is differential expression of BM proteins in normal and malignant prostate cells [54,126]. In fact, loss of BM continuity has been correlated with PCa malignancy [127]. It has also been reported that malignant PCa cells lack the γ2 sub-chain of laminin-5 (laminin-332) [51,52,54]. This loss of laminin-5 function may occur only in PCa, despite being highly expressed in other cancers, such as colon and breast carcinomas [51]. This has been suggested to contribute to accelerated migratory behavior in PCa [53], suggesting that the status of the BM is important for metastasis. It is also interesting to note that this loss in laminin-5 is concurrent with the activation of Shh signaling during progression of PCa. It has long been established that Shh is detected in metastasizing PCa, regulating the signaling pathways for proliferation and invasion [128]. In fact, overexpressing Shh can transform normal prostate cells into metastatic pCa cells [129]. There appears to be a possibility that Shh promotes PCa metastasis by affecting the BM condition via laminin-5. Whether these observations can be applied to the varying conditions of PCa in vivo should be studied further. Thus, the involvement of Hh signaling in the regulation of the BM during PCa formation or progression has yet to be established. Further studies are necessary to investigate the possible correlations of abnormal cell migration to the status of the BM in mutant mice. Alteration of actions of growth factors and hormones and cellular behaviors across the affected BM region should be investigated further (Table 1, Figure 1).

Modulating Hh signal may lead to altered laminin production in the BM. Matrix metalloproteinases (MMPs), which can cleave extracellular matrix proteins, have been suggested to be involved in tumorigenesis in several cancers, including PCa [130,131]. Whether the correlation of such enzymes with abnormal distribution of proteins is possible and requires further analysis. It has been reported that Shh signaling can regulate the expression of MMPs, leading to accelerated migration in ovarian, liver, and gastric cancers [132,133,134]. Hence, Hh signaling may also indirectly affect the status of the BM via MMP regulation.

## 6. Conclusions

EMI (epithelial–mesenchymal interaction) is a fundamental concept which governs both organogenesis and cancer progression. In this text, we use the development of the EXG and prostate cancer as representatives of these events. Hh signaling has been shown to play a vital role in both processes, and it is worth noting that the effect of Hh signaling might either be positive or detrimental depending on the tissue and developmental context. This review offers a summary of the possible and known roles of Hh signaling during EMI. Although there are several other signaling pathways by which Hh can interact, we show here the importance of the Hh/Bmp signaling interactions in the two systems. This crosstalk is necessary for the proper development of the urogenital system and is also responsible for driving part of PCa malignancy. Furthermore, Shh can induce local steroidogenesis of androgens, a hormone that is critical for both events of interest. This local action is currently a concern as it offers possible therapeutic targets for PCa. Lastly, we discussed the potential role of the BM during EMI. The BM has long been believed to function as a barrier between the epithelia and the mesenchyme, but we describe that Hh can participate in EMI via the BM. Insights from the role of Hh in each biological event may contribute further understanding of this complex signaling pathway. Studying this pathway using comparative and interdisciplinary viewpoints would be necessary to understand even better the Hh signaling function and its implications in EMI.

## Figures and Tables

**Table 1:** Representative references showing various mechanisms and functions of epithelial-mesenchymal interactions (EMI) in organogenesis and tumorigenesis (shown by blue color). Hormonal signal has been also reported as locally produced and their actions are cited in the table. Representative EMI processes include hedgehog ligands, interacting growth factor (BMP: bone morphogenetic protein)-Bmp receptor, local hormones (steroidogenesis) and basement membrane (BM). Yellow colored columns represent works showing the crosstalks for hedgehog (Hh)/Bmp and Hh-steroidogenesis. Two papers are marked with parentheses for the roles of BM in EMI.

		Prostate Cancer Progression	Exg Organogenesis
**Epithelial–Mesenchymal Interaction (EMI)**	Hedgehog Signaling		
	Ligands	*Shh* (*Sonic hedgehog*) Fan et al., 2004, *Endocrinology* [35] Wilkinson et al., 2013, *Prostate* [36] Nishimori et al., 2012, *J Biol Chem* [37]	*Shh* Haraguchi et al., 2001, *Development* [19] Haraguchi et al., 2007, *Development* [22]
	Mediators	*Gli* Sanchez et al., 2004, *PNAS* [38] Chen et al., 2010, *Mol cancer* [39]	*Gli* Miyagawa et al., 2011, *Endocrinology* [40] He et al., 2016, *PLoS One* [16]
	Hh/Bmp Crosstalk	Shaw et al., 2010, *Differentiation* [41]	Haraguchi et al., 2012, *PLoS One* [27]
	Bmp Signaling		
	Bmp Ligands	*BMP2* Horvath et al., 2004, *Prostate* [42] *BMP4* Lee at al., 2011, *Cancer Res* [43] *BMP7* Masuda et al., 2003, *Prostate* [44]	*BMP4* Kajioka et al., 2019, *Congenit Anom* [24] Ching et al., 2018, *Hum Mol Genet* [28] *BMP7* Suzuki et al., 2008, *Eur J Hum Genet* [26]
	Bmp Receptor	Ide et al., 1997, *Oncogene* [45] Kim et al., 2000, *Cancer Res* [46]	Suzuki et al., 2003, *Development* [23]
	Paracrine Action of Local Steroidogenesis	Lubik et al., 2016, *Int J Cancer* [47] Levina et al., 2012, *prostate* [48]	Suzuki et al., 2017, *Andrology* [20]
		*Backdoor production of dihydrotestosterone* (*DHT*) Chang et al., 2011, *PNAS* [49]	*Backdoor production of DHT* O’Shaughnessy et al., 2019, *PLoS Biol* [50]
	Role of the BM	Davis et al., 2001, *Prostate* [51] Hao et al., 1996, *Am J Pathol* [52] Hao et al., 2001, *Am J Pathol* [53] Nagle, 2003, *J Biol Chem* [54]	Lin et al., 2016, Mech Dev [55] Gao et al., 2008, *Genes Dev* [56] Pickering et al., 2017, *Matrix Biol* [57]

## Abbreviations

Hh	hedgehog
EMI	epithelial–mesenchymal interactions
EXG	external genitalia
PCa	prostate cancer
BMP	bone morphogenetic protein
Shh	Sonic hedgehog
Ihh	Indian hedgehog
Dhh	Desert hedgehog
Ptch	Patched
Smo	Smoothened
GT	genital tubercle
PSA	prostate specific antigen
CAF	cancer associated fibroblasts
DHT	dihydrotestosterone
CRPC	castration-resistant prostate cancer
AR	androgen receptor
BM	basement membrane
MMP	matrix metalloproteinases
